# Treatment of Sleeplessness

**Published:** 1878-04

**Authors:** 


TREATMENT OF SLEEPLESSNESS.—When sleep is broken by severe pain, opium or morphia is of value, not only by relieving the pain but by its action in producing anæmia of the cerebral vessels. In the wakefulness due to neuralgia, it is often better to inject a small dose of morphia hypodermically near the branch of the affected nerve then to administer it by the mouth. When the sleeplessness arises from the pains of muscular spasm (as in gallstone colic), or is attended with headache, flushing of the face, and other symptoms of cerebral hyperæmia, chloral-hydrate is indicated, as this is supposed to owe its hypnotic effect to its power of diminishing the amount of blood in the brain. On the other hand, in wakefulness due to defective cardiac power, digitalis is often useful by strengthening the action of the heart and relieving the congestion of the central organs and the anæmia of the extremities. It is doubtful whether the bromides possess hypnotic properties, although they undoubtedly act as sedatives on the nervous system, and as such may occasionally induce sleep.—*Boston Jour. of Chemistry.*

